# The Impact of Tyrosine Kinase Inhibitors on Fatherhood in Patients With Chronic Myeloid Leukemia: A Mixed-Method Study

**DOI:** 10.7759/cureus.33407

**Published:** 2023-01-05

**Authors:** Mohammad Abu-Tineh, Elrazi A Ali, Awni Alshurafa, Abdulqadir J Nashwan, Khalid Albsheer, Ashraf Ahmed, Yousef Hailan, Waail Rozi, Esraa Aljaloudi, Mohamed A Yassin

**Affiliations:** 1 Department of Medical Oncology, Hematology and BMT Section, National Center for Cancer Care and Research, Doha, QAT; 2 Internal Medicine, One Brooklyn Health / Interfaith Medical Center, Brooklyn, USA; 3 Nursing Department, Hamad Medical Corporation, Doha, QAT; 4 Internal Medicine, Hamad General Hospital, Doha, QAT; 5 Internal Medicine, Hamad Medical Corporation, Doha, QAT; 6 Department of Family Medicine, Hamad Medical Corporation, Doha, QAT

**Keywords:** fatherhood, chronic myeloid leukemia, cml, tyrosine kinase inhibitors, tkis

## Abstract

Introduction: Multiple studies have demonstrated that tyrosine kinase inhibitors (TKIs) exert a significant extent of control over chronic myeloid leukemia (CML), as evidenced by studies such as the population-based Swedish CML registry, which found that patients reaching age 70 had a relative survival rate close to one when compared to the general population. Consequently, new perspectives on the safety of treatments have emerged, particularly in the context of their impact on fatherhood in men. According to the authors, this is the first study to examine the effect of TKIs on fatherhood in CML patients.

Methods: A single-center, mixed-design study (retrospective data review and phone interviews) was conducted with CML male patients in the chronic or accelerated phase, evaluating the effect of imatinib, dasatinib, and nilotinib on their fatherhood, irrespective of whether they were administered as a first, second, or third line of treatment.

Results: The study included interviews with 150 patients. Included were 27 patients. The average age was approximately 44.5 years. One hundred percent of the patients were in the chronic phase. The median age at first conception following TKI therapy was 36, and the median duration of TKI therapy was approximately seven years. The total number of offspring was 49; 98% were born at term and had a normal birth weight. No reports of stillbirths, fetal deaths, or congenital malformations were made. All the offspring grew and developed normally. No CML-related cancers were reported in any of the newborns.

Conclusion: Around 98% of male CML patients receiving imatinib, dasatinib, or nilotinib did not experience a negative impact on their fatherhood or the health of their children. However, improved education for patients beginning treatment with TKIs addresses the potential psychological worry of having an unfavorable effect on their fertility or offspring, which may increase medication adherence.

## Introduction

Chronic myeloid leukemia (CML) is a clonal myeloproliferative neoplasm characterized by the unregulated proliferation of myeloid cells in the bone marrow with the presence of a reciprocal translocation between chromosomes 9 and 22, or the Philadelphia chromosome (Ph), causing the formation of the BCR-ABL fusion gene, encoding the oncogenic tyrosine kinase BCR-ABL [1]. The median age for CML patients in Western countries is about 57 years, with patients older than 70 being around 20% and adolescents and children being <5%; however, in Africa and Asia, the median age of diagnosis is <50, which reflects the low median age of the population, with slightly more men than women [2,3]. 

Patients with CML are in one of the three main disease phases: the chronic phase, the accelerated phase, or the blast crisis. Patients can progress to other phases as their disease responds or does not respond to treatment [4]. The treatment options for patients with CML are variable. The choice of treatment depends on multiple factors, such as the phase of CML, the availability of a donor for hematopoietic cell transplantation (HCT), patient age, and the presence of medical comorbidities affecting patient applicability for HCT or specific tyrosine kinase inhibitors (TKIs). The TKIs, particularly imatinib, are the initial treatment of choice for most patients with chronic phase CML [5]. Response to therapy and the need to use second-line therapy are usually checked regularly based on the hematologic, cytogenetic, and molecular levels.

The efficacy of TKIs for treating CML has been universally accepted, with patients seeking better living standards, including fertility and family planning. Pye et al. [6] reviewed the outcome of 180 women exposed to imatinib during pregnancy, followed by another large study [7] involving 265 female patients taking imatinib, with results corroborating that the majority of pregnancies occurring during imatinib therapy may indeed have a successful outcome. Nevertheless, there are a considerable number of drug-related serious fetal malformations and a somewhat higher risk of spontaneous abortion. Therefore, pregnancy should not be avoided but planned. However, despite multiple studies which have evaluated the effect of TKIs on the reproductive system in female patients and their pregnancy outcomes, and only a few studies looking into the impact of TKIs on the spermatogenesis of male patients with CML [1,8], the concept is still controversial with limited data available worldwide. To the authors' knowledge, this is the first study addressing the impact of TKIs on the fatherhood of CML male patients. This article was previously presented as an abstract at the 2021 American Society of Hematology meeting on December 11, 2021. 

## Materials and methods

Methods

This is a mixed-design study conducted by both retrospective data review and phone interviews with CML male patients in the chronic or accelerated phase, being followed up at the National Center for Cancer Care and Research (NCCCR), evaluating the effect of three types of TKIs on their fertility if they are taking them as a first, second, or third line of treatment.

All patients were treated in our center between January 1, 2005, and January 1, 2020. Aged 18 years old and older, they agreed to be interviewed by phone. All data was stored, coded, and anonymized on a hospital computer in the medical oncology department at NCCCR, Hamad Medical Corporation (HMC), and secured by a designated HMC username and password. Data could be accessed only by the research team. One hundred and fifty patients were included in the study. Hamad Medical Corporation Institutional Review Board, The Medical Research Center (MRC) issued approval MRC-01-20-418.

Participants, study population and study setting/location

A total of 150 patients were interviewed, along with data and records from the NCCCR.

Adult male patients aged ≥18 years old, diagnosed with CML (chronic or accelerated) phase, who were currently receiving imatinib, nilotinib, or dasatinib as the first, second, or third line of treatment, according to the European Leukemia Network (ELN) for CML diagnosis and staging.

Initial TKI dose: 400 mg per day for imatinib, 600 mg per day for nilotinib, and 100 mg per day for dasatinib. Second-line dosing: 300 mg twice daily or 600 mg once daily for imatinib, 400 mg twice daily for nilotinib, 100 mg daily, or 140 mg daily for dasatinib.

All patients reside in Qatar, with regular follow-ups at the NCCCR.

Inclusion criteria

Male patients, 18 years or older, who have been diagnosed with chronic or accelerated CML and were receiving tyrosine kinase inhibitors, including imatinib, dasatinib, and nilotinib, had the following: patients with intact fertility or patients with an andrologist-identified TKI-related fertility issue after starting TKIs.

Exclusion criteria

Patients with other myeloproliferative neoplasms (MPNS) and CML patients who did not meet the inclusion criteria, such as those infertile before their CML diagnosis or who became infertile after their CML diagnosis but whose underlying cause was clearly unrelated to TKIs, were excluded. Patients with infertility with no evaluation done by an andrologist to identify it as TKI-related or if the wife had a gynecologist-verified infertility issue

Outcomes

To evaluate the effects of TKIs on fatherhood in male patients diagnosed with CML.

## Results

**Figure 1:**
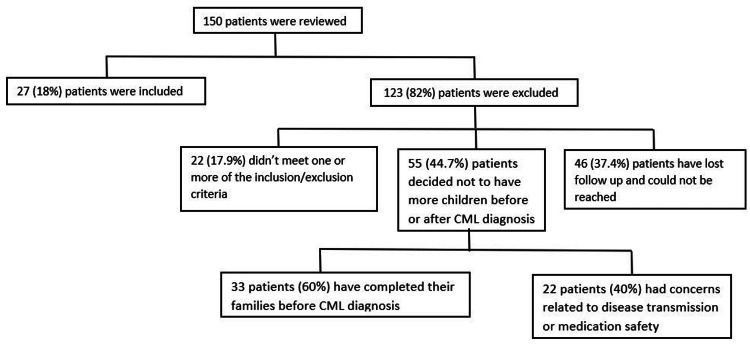
The number of patients included and excluded from the study. CML: Chronic Myeloid Leukemia

A total of 150 patients were reviewed. One hundred twenty-three (82%) of the patients had no offspring during the treatment period, given the older average age of the CML diagnosis. Most of the patients decided not to have more children because they had completed their families before diagnosis or had concerns about disease transmission or medication safety. Other patients have lost medical follow-up, especially in Qatar, a country with many expatriates who travel and change frequently (Figure 1).

Twenty-seven (18%) male patients were included in the analysis. All were in the chronic phase. The median age of the patients was 44.5 years (range: 28-66), and the median age at diagnosis was 34 (range: 16-56). All patients were receiving TKI: 12 (44.4%) with imatinib, nine (33.3%) with nilotinib, and six (22.2%) with dasatinib. The median duration of treatment was seven years (range: 1-21). All patients were compliant with and tolerant of TKI therapy with regular hematology follow-ups. The median age of these male patients at the time of the first conception was 36 years (range, 24-62 years), and the duration of TKI exposure before the first pregnancy was three years (range, 1-9 years).

The total number of conceptions was 50. One abortion was reported, and the other 49 pregnancies had successful deliveries, with 45 (91.8%) normal vaginal deliveries and four (8.2%) with a cesarean section. Only one of the 49 children was born prematurely in week 33 as the first child and was underweight, while the others were full-term and of normal weight. No stillbirth or intrauterine fetal demise was reported. Figure 1 and Table 1 show that the median age of these 49 children was seven years (range: 1-16). All children had normal growth and development, with no reports of cancer-related or unrelated myeloproliferative neoplasms till the data collection.

**Table 1:** Characteristics of patients included in the study CML: Chronic Myeloid Leukemia, TKI: Tyrosine Kinase Inhibitors

Table 1: patients characteristics (N=27)	
Age in years, median	44.5
Age Range	(28-66)
Age at diagnosis, median	34
Age at diagnosis Range	(16-56)
CML phase:	
Chronic	27 (100 %)
Accelerated	0
TKI treatment:	
Imatinib	12 (44.4 %)
Nilotinib	9 (33.3 %)
Dasatinib	6 (22.2 %)
Duration of TKI treatment, median	7
Range	(1-21)
Patients' age at the time of the first conception, median	36
Range	(24-62)
TKI exposure before first pregnancy, median	3
Range	(1-9)
Total number of conceptions	50
Number of abortions	1 (2%)
Mode of delivery:	
Normal vaginal delivery	45 (91.8 %)
Cesarian section	4 (8.2 %)
Stillbirth or intrauterine birth demise	0
Age of born children after CML diagnosis (median)	7
Range	(1-16)

## Discussion

The ability to conceive and give birth to a child is referred to as "fertility." It refers to a woman's capacity to become pregnant and carry a child during pregnancy through normal sexual activity. Men's fertility refers to their ability to father a child through sexual activity. Fertility is influenced by a variety of factors, including a person's reproductive organs and several other factors, such as when and how often they have sex, hormone levels, and whether their partner has fertility concerns [9]. Cancer can impair fertility directly by affecting reproductive organs and indirectly by inhibiting reproductive function or delaying reproduction due to cancer treatment.

Moreover, patients with CML may develop priapism like other MPNs, which can affect their fertility, although none of our included patients reported priapism [10-11]. Pregnancy rates among women over 35 have grown in the last 25 years. As a result, more cancer patients are diagnosed and treated when their reproduction is incomplete, raising the importance of reproductive difficulties. Cancer survivors' reproductive loss is significant, and their fertility concerns are frequently unaddressed [9]. Infertility is linked to severe psychological suffering, with depression levels twice those of the general population and a reduction in quality of life in areas such as emotional well-being, relationships, and sexuality. When infertility is combined with cancer, the patient, spouse, and family are under so much burden. Even for those who do not intend to have children, fearing infertility can leave them feeling bereft and angry [12].

Specialized fertility psychological interventions and counseling may be beneficial in alleviating patient distress, assisting treatment decision-making processes, and providing necessary reproductive education when cancer or cancer treatment impacts fertility potential, according to the literature [13].

Chronic myeloid leukemia is a myeloproliferative neoplasm characterized by the anomalous amplification of the myeloid compartment of the hematopoietic system. The hallmark of the disease is the presence of a reciprocal translocation between the long arms of chromosomes 9 and 22, t(9;22) (q34;q11.2), causing a derivative 9q+ and a small 22q- [14]. The Philadelphia (Ph) chromosome results in a BCR-ABL fusion gene and the production of a BCR-ABL fusion protein [15]. BCR-ABL encodes a constitutively active protein, tyrosine kinase, which is required to develop the disease [14]. The appearance of this well-defined defect at the molecular level was the cornerstone for developing TKIs, initially imatinib, later dasatinib, and nilotinib. All TKIs block the BCR-ABL aberrant molecule, shutting down the leukemia phenotype [7]. 

Currently, due to the marked outcome of controlling CML with TKIs, there is an increased desire for parenting a child for most patients (men and women) of childbearing age who still have not completed their families. Physicians are usually asked for advice regarding whether to hold or stop the treatment before conception. Unfortunately, the data relating to the safety of the TKI before, during, and after gestation remains controversial. Most information regarding the reproductive effects of imatinib is derived from preclinical animal studies. In a rat model, high doses of imatinib increased embryonic loss and malformations (exencephaly, encephalocele, and chemical bone hypoplasia) [16-17]. Imatinib inhibits not only BCR-ABL, the putative cause of CML, but also other tyrosine kinases, such as c-ABL, c-KIT, platelet-derived growth factor receptor (PDGFR), and ARG [18]. Nilotinib, a second-generation TKI, also inhibits these proteins known to have a function that may be important in gonadal development, implantation, and fetal development, hence the safety of TKI on conception should be considered. For obvious reasons, the effects of TKI on fertility, pregnancy, and lactation remain quite limited. Most of the information was derived primarily from animal experiments and case reports. Therefore, clinical observations are very important [19]. Some have suggested that imatinib is unlikely to cause significant fetal abnormalities when given during the third trimester of pregnancy due to poor transport across the placenta [16,20]. The published data on pregnancy outcomes in patients who receive TKIs for Philadelphia-positive CML and other lymphoproliferative disorders is mostly based on a few case reports or case series and thus cannot be extrapolated to a larger cohort [18,20-24]. 

Later, more extensive studies [6,19,21,25] reported on around 61 and 18 female patients who conceived while being treated with imatinib for CML [21], with an outcome of over 80% of pregnancies being uneventful and children having normal milestones. However, although multiple studies address the effects of TKIs on pregnancies in female patients, the data on the impact of TKIs on the reproductive system in male patients with CML is still limited. Two studies [1,26] that looked at male spermatogenesis found that serum levels of testosterone (T), luteinizing hormone (LH), and follicle-stimulating hormone (FSH) all dropped by a significant amount.

A very limited number of studies worldwide have addressed the outcome of male patients with CML on TKIs [8,27-30]. But the lack of data is still concerning regarding the fatherhood of male patients diagnosed with CML. Our study screened 150 patients, with 27 male patients fitting the inclusion criteria. The data had an almost similar outcome to a recent review [30-31], as our study had shown a 2% chance of abortion. In addition, 100% of offspring had normal growth and development with 0% incidence of blood-related diseases. Data suggests the limited effect of TKIs on male fatherhood outcomes and the well-being of their offspring. However, further studies with a larger sample size are needed to study the TKIs' effects on fatherhood in CML patients. Moreover, there is a need for enhanced education for patients starting TKIs, highlighting the possible psychological fears or concerns about having an unfavorable effect on their fertility or offspring for better medication acceptability and adherence.

## Conclusions

The study concluded that 98% of male patients on TKI for CML (imatinib, dasatinib, or nilotinib) remained fertile and had healthy kids with no congenital defects, impaired growth, or malignancies.

## References

[1] Yassin MA, Soliman AT, Sanctis VD (2014). Effects of tyrosine kinase inhibitors on spermatogenesis and pituitary gonadal axis in males with chronic myeloid leukemia. J Cancer Res Ther.

[2] Hochhaus A, Baccarani M, Silver RT (2020). European LeukemiaNet 2020 recommendations for treating chronic myeloid leukemia. Leukemia.

[3] Turkina A, Wang J, Mathews V (2020). TARGET: a survey of real-world management of chronic myeloid leukaemia across 33 countries. Br J Haematol.

[4] Cortes JE, Talpaz M, O'Brien S (2006). Staging of chronic myeloid leukemia in the imatinib era: an evaluation of the World Health Organization proposal. Cancer.

[5] O'Brien SG, Guilhot F, Larson RA (2003). Imatinib compared with interferon and low-dose cytarabine for newly diagnosed chronic-phase chronic myeloid leukemia. N Engl J Med.

[6] Pye SM, Cortes J, Ault P (2008). The effects of imatinib on pregnancy outcome. Blood.

[7] Abruzzese E, Trawinska MM, Perrotti AP, De Fabritiis P (2014). Tyrosine kinase inhibitors and pregnancy. Mediterr J Hematol Infect Dis.

[8] Chang X, Zhou L, Chen X (2017). Impact of imatinib on the fertility of male patients with chronic myelogenous leukaemia in the chronic phase. Target Oncol.

[9] Penrose R, Beatty L, Mattiske J, Koczwara B (2013). The psychosocial impact of cancer-related infertility on women: a review and comparison. Clin J Oncol Nurs.

[10] Ali E, Soliman A, De Sanctis V, Nussbaumer D, Yassin M (2021). Priapism in patients with chronic myeloid leukemia (CML): a systematic review. Acta Biomed.

[11] Ali EA, Nashwan AJ, Yassin MA (2021). Essential thrombocythemia with (type2) calreticulin presented as stuttering priapism case report and review of literature. Clin Case Rep.

[12] Carter J, Rowland K, Chi D, Brown C, Abu-Rustum N, Castiel M, Barakat R (2005). Gynecologic cancer treatment and the impact of cancer-related infertility. Gynecol Oncol.

[13] Logan S, Perz J, Ussher JM, Peate M, Anazodo A (2019). Systematic review of fertility-related psychological distress in cancer patients: informing on an improved model of care. Psycho-oncology.

[14] Thompson PA, Kantarjian HM, Cortes JE (2015). Diagnosis and treatment of chronic myeloid leukemia in 2015. Mayo Clin Proc.

[15] Shtivelman E, Lifshitz B, Gale RP, Canaani E (1985). Fused transcript of abl and bcr genes in chronic myelogenous leukaemia. Nature.

[16] Mukhopadhyay A, Dasgupta S, Kanti Ray U, Gharami F, Bose CK, Mukhopadhyay S (2015). Pregnancy outcome in chronic myeloid leukemia patients on imatinib therapy. Ir J Med Sci.

[17] Russell MA, Carpenter MW, Akhtar MS, Lagattuta TF, Egorin MJ (2007). Imatinib mesylate and metabolite concentrations in maternal blood, umbilical cord blood, placenta and breast milk. J Perinatol.

[18] (2022). GLEEVEC® (imatinib mesylate). https://www.novartis.com/us-en/sites/novartis_us/files/gleevec_tabs.pdf.

[19] Zhou L, You JH, Wu W, Li JM, Shen ZX, Wang AH (2013). Pregnancies in patients with chronic myeloid leukemia treated with tyrosine kinase inhibitor. Leuk Res.

[20] Eskander RN, Tarsa M, Herbst KD, Kelly TF (2011). Chronic myelocytic leukemia in pregnancy: a case report describing successful treatment using multimodal therapy. J Obstet Gynaecol Res.

[21] Iqbal J, Ali Z, Khan AU, Aziz Z (2014). Pregnancy outcomes in patients with chronic myeloid leukemia treated with imatinib mesylate: short report from a developing country. Leuk Lymphoma.

[22] Goel N, Malik R, Rathi B, Bhaskaran S, Rajaram S, Mehta S, Agarwal N (2013). Pregnancy with metastatic gastrointestinal stromal tumor (GIST) on imatinib chemotherapy: an oncologist's nightmare and obstetrician's dilemma. J Gastrointest Cancer.

[23] Tsuzuki M, Inaguma Y, Handa K (2009). Successful pregnancy in a patient with chronic myeloid leukemia under treatment with imatinib. Intern Med.

[24] Conchon M, Sanabani SS, Bendit I, Santos FM, Serpa M, Dorliac-Llacer PE (2009). Two successful pregnancies in a woman with chronic myeloid leukemia exposed to nilotinib during the first trimester of her second pregnancy: case study. J Hematol Oncol.

[25] Ault P, Kantarjian H, O'Brien S (2006). Pregnancy among patients with chronic myeloid leukemia treated with imatinib. J Clin Oncol.

[26] Seshadri T, Seymour JF, McArthur GA (2004). Oligospermia in a patient receiving imatinib therapy for the hypereosinophilic syndrome. N Engl J Med.

[27] Shash E, Bassi S, Cocorocchio E, Colpi GM, Cinieri S, Peccatori FA (2011). Fatherhood during imatinib. Acta Oncol.

[28] Ramasamy K, Hayden J, Lim Z, Mufti GJ, Ho AY (2007). Successful pregnancies involving men with chronic myeloid leukaemia on imatinib therapy. Br J Haematol.

[29] Dou XL, Qin YZ, Shi HX, Lai YY, Hou Y, Huang XJ, Jiang Q (2019). [Fertility and disease outcomes in patients with chronic myeloid leukemia]. Zhonghua Xue Ye Xue Za Zhi.

[30] Szakács Z, Hegyi PJ, Farkas N (2020). Pregnancy outcomes of women whom spouse fathered children after tyrosine kinase inhibitor therapy for chronic myeloid leukemia: a systematic review. PLoS One.

[31] Abu-Tineh M, Ali EA, Alshurafa A (2021). The impact of tyrosine kinase inhibitors on fatherhood in patients with chronic myeloid leukemia, a single institution experience. Blood.

